# Induction of Apoptosis in Endometrial Cancer (Ishikawa) Cells by *Pogostemon cablin* Aqueous Extract (PCAE)

**DOI:** 10.3390/ijms160612424

**Published:** 2015-06-02

**Authors:** Ching-Chou Tsai, Ya-Huei Chang, Chi-Chang Chang, Ya-Min Cheng, Yu-Che Ou, Chan-Chao Chang Chien, Yi-Chiang Hsu

**Affiliations:** 1 Department of Obstetrics and Gynecology, Chang Gung Memorial Hospital, Kaohsiung 83301, Taiwan; E-Mails: nick@adm.cgmh.org.tw (C.-C.T.); ou4727@cloud.cgmh.org.tw (Y.-C.O.); chanchao@cgmh.org.tw (C.-C.C.C.); 2 Innovative Research Center of Medicine, College of Health Sciences, Chang Jung Christian University, Tainan 71101, Taiwan; E-Mail: yahuei1223@gmail.com; 3 Graduate Institute of Medical Science, College of Health Sciences, Chang Jung Christian University, Tainan 71101, Taiwan; 4 Department of Obstetrics and Gynecology, E-Da Hospital, E-Da Hospital/I-Shou University, Kaohsiung 82445, Taiwan; E-Mail: p2696373@yahoo.com.tw; 5 Department of Obstetrics and Gynecology, Institute of Clinical Medicine, College of Medicine, National Cheng Kung University, Tainan 71701, Taiwan; E-Mail: chengym@mail.ncku.edu.tw

**Keywords:** apoptosis, endometrial cancer, *Pogostemon cablin* aqueous extract (PCAE)

## Abstract

*Pogostemon cablin* (PC) is a traditional herbal medicine used in the treatment of the common cold, nausea, diarrhea, and even for headaches and fever. However, the mechanisms underlying the anti-proliferative activity of PC in endometrial cancer (EC) cells have yet to be fully elucidated. This study investigated the anticancer effects of an aqueous extract of *Pogostemon cablin* (PCAE), specifically induced apoptosis in EC (Ishikawa) cells. Proliferation of EC cells following exposure to PCAE was assessed by an MTT assay. DNA content and the induction of cell cycle apoptosis were analyzed by flow cytometry (FACS Calibur). Protein caspase-3 and, -9 as well as AIF were investigated using Western blot. Our results demonstrate growth inhibition of Ishikawa cells by PCAE. Furthermore, caspase-3 activity caused PCAE-treated cell lines to accumulate in apoptosis. Gene expression profiling (GEP) results further suggest that, in addition to its known effects with regard to EC prevention, PCAE may also exert antitumor activity on established EC cells. Many previous studies have identified the chemo-preventive effects of natural plant materials and the potential role of these materials in chemotherapy. This current study used human EC Ishikawa cells to investigate the anti-tumor effects of PCAE in EC cells. Our results demonstrate that PCAE inhibits the growth of cancer cells and induces apoptosis, which suggests the potential applicability of PCAE as an antitumor agent.

## 1. Introduction

The prevalence of endometrial cancer (EC) tends to be higher in developing countries [1]; however, the number of new cases is increasing worldwide. In recent decades, the reported incidence of EC in developing countries has decreased due to improvements in screening as well as changes in dietary habits and lifestyle. Increases in malignancy have forced physicians to develop new diagnostic and treatment modalities for this cancer [2]. Despite the fact that the etiology of EC is not yet clearly understood, epidemiological studies have observed a positive relationship between obesity and the risk of EC [3]. EC is classified into stages according to microscopic patterns and its ability to invade uterine muscle, which also determines the risk of recurrence and largely determines treatment options. The most common pathological type is an endometrioid adenocarcinoma; however, approximately 10% of endometrial cancer cases present a clear cell histologic appearance [4].

Severe side effects and limits to the therapeutic efficacy of conventional cancer treatments has led to the widespread application of complementary and/or alternative medicines (CAMs) [5]. Among the various treatment modalities, traditional medicines with demonstrable anti-tumor activity are the preferred choice [6]. *Pogostemon cablin* (PC) in tien-hsien liquid (THL) is a Chinese herbal mixture that has been used as a complementary anticancer agent for more than 10 years [7]. THL has recently been shown to induce apoptosis in cancer cells and activate caspase-3, -8, and -9 [8]. The biological activities of PC have been widely reported, including effects related to antioxidation and antimutagenesis [9,10]. In addition, the effects of PC on phlegm elimination are well-known in traditional medicine, which has led to PC being commonly prescribed in conjunction with apoplexy therapy and syncope with eating or drinking [11]. Finally, PC has also proven beneficial in the treatment of cerebral stroke [12]. Despite the fact that many of these studies provided information related to the isolation and identification of PCAE [13] and molecular basis of PC-induced anticancer activities, the underlying mechanisms have yet to be fully elucidated. In the current study, we explored the effects of PCAE on EC (Ishikawa) cells in order to investigate the underlying molecular mechanisms. Apoptosis and cell-cycle regulation have been suggested as targets for cancer therapy [9,14]. There may also be a close link between PCAE-induced apoptosis and regulation of cell cycle progression. We therefore specifically focused on the influence of PCAE on the induction of apoptosis and apoptosis-related gene expression in Ishikawa cells.

## 2. Results and Discussion

### 2.1. PCAE Inhibits the Cell Survival and Proliferation of Ishikawa Cells

**Figure 1:**
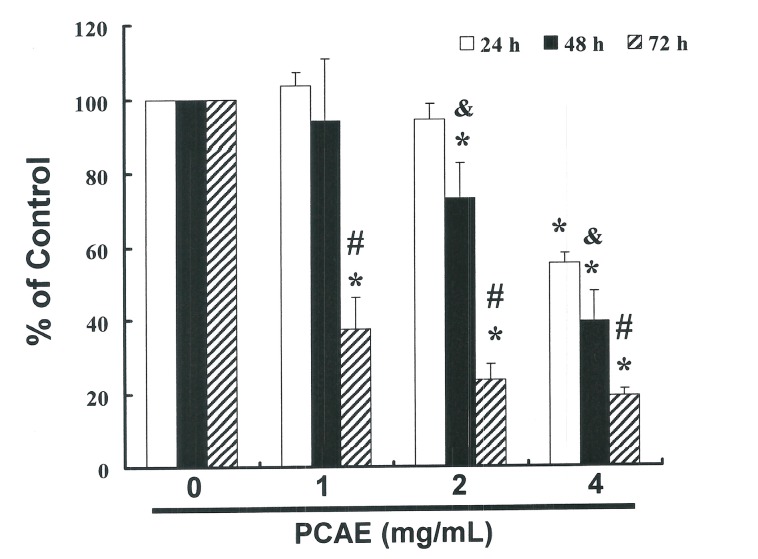
PCAE mediates the survival of EC cells through the inhibition of proliferation. Ishikawa cells were treated with increasing doses of PCAE (0, 1, 2 and 4 mg/mL) for 24 to 72 h *in vitro*. The survival of PCAE-treated cancer cells was measured using the MTT method. Results are expressed as a percentage of the control, which was considered to be 100%. All data are reported as the mean (±SEM) of at least three separate experiments. Statistical analysis was performed using a *t*-test, with significant differences (*p* < 0.05) between treatment and control (PCAE 0 mg/mL) groups, and & *vs.* the 24 h group and # *vs.* the 48 h group, delineated by the * symbol.

This study hypothesized that PCAE could mediate the survival of Ishikawa cells and thereby inhibit proliferation. The anti-tumor activity of PCAE against Ishikawa cells was investigated *in vitro* by treating Ishikawa cells with increasing doses of PCAE (0, 1, 2, and 4 mg/mL) over a period of 24 to 72 h. We then measured the proliferation of PCAE-treated cancer cells using the MTT method, the results of which are summarized in Figure 1. Our findings indicate that the survival and proliferation of Ishikawa cells decreased with an increase in the dose of PCAE (*y* = −14.306 *x* + 124.02 *R*^2^ = 0.6902). A microscopic examination further revealed morphological changes in Ishikawa cells following exposure to PCAE (2 mg/mL) for 6 to 24 h, and PCAE was also shown to induce the death of cancer cells, which resulted in a suspension of dead cells in the medium (data not shown).

### 2.2. PCAE-Induced Apoptosis of Ishikawa Cells

**Figure 2:**
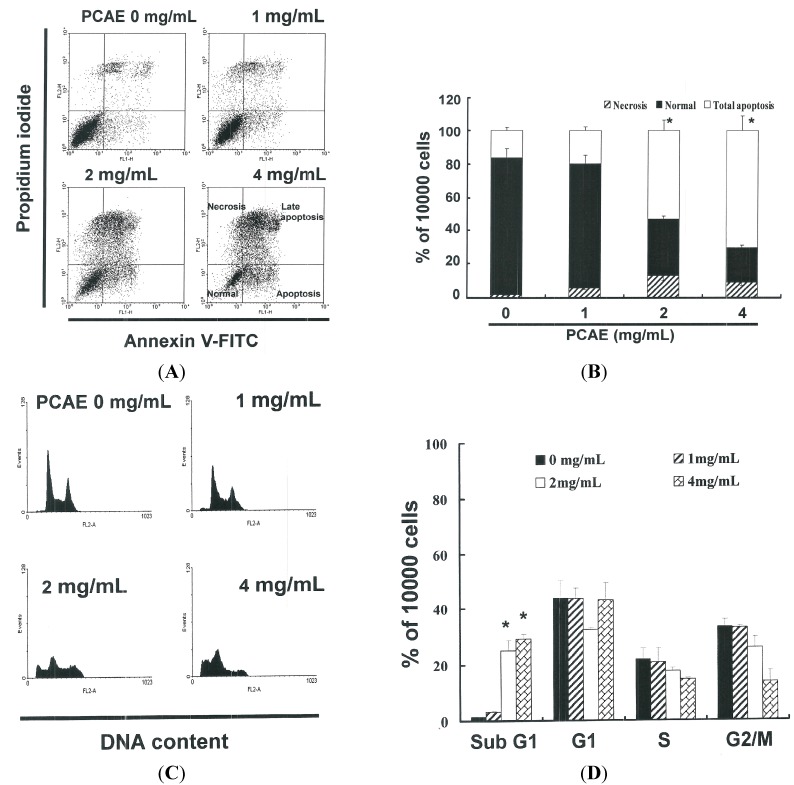
(**A**) The influence of PCAE on apoptosis/necrosis in Ishikawa cells; (**B**) Total apoptosis in Ishikawa cells following incubation with PCAE for 4 h. (* *p* < 0.05 *vs.* PCAE 0 mg/mL control group). Influence of PCAE on cell cycle progression/distribution in Ishikawa cells: (**C**) cell cycle analysis of Ishikawa cells after being cultured with PCAE for 24 h; and (**D**) PCAE induced an increase in sub G_1_ (%). Cells underwent dual staining using propidium iodide to analyze DNA content, which was then quantified using flow cytometry. The * symbol in each group of bars indicates that the number of sub G_1_ cells in the PCAE treatment group was significantly greater than that of the control at a significance level of *p* < 0.05.

The ApopNexin FITC apoptosis detection kit was used to identify apoptotic Ishikawa cells following exposure to PCAE for 6 h. PI-annexin-V double staining was used to differentiate intact cells from early apoptotic cells, late apoptotic cells, and dead (necrotic) cells as well as to investigate apoptosis in greater detail. Typical assay results are presented in Figure 2A, in which annexin V-FITC deposits indicate the existence of apoptotic cells. As shown in Figure 2B, apoptosis was induced in a dose-dependent manner when administered at 2 and 4 mg/mL (*y* = 19.72 *x* − 9.33 *R*^2^ = 0.9265). Increases of the percentages of apoptotic Ishikawa cells was observed at all dose levels following treatment for 6 h, wherein approximately 16.17% ± 2.02% of the Ishikawa cells were in early or late stages of apoptosis. The percentage of apoptotic Ishikawa cells increased to 19.73% ± 2.46% under PCAE treatment of 1 mg/mL. When the PCAE concentration was increased to 2 and 4 mg/mL, the percentage apoptotic Ishikawa cells increased to 53.25% ± 6.66% and 70.73% ± 8.84%, respectively. Taken together, these observations strongly imply that PCAE significantly elevated apoptosis in EC cells.

### 2.3. PCAE Treatment Led to Accumulation of Sub G_1_ Ishikawa Cells

The cell-cycle distribution of PCAE-treated Ishikawa cells was analyzed using flow cytometry, with the aim of determining whether inhibitory effects were due to cell-cycle arrest or apoptosis. Prior to processing and analysis, the cells were exposed to PCAE for 24 h. As shown in Figure 2C, the samples exposed to PCAE included a larger number of cells in the sub G_0_/G_1_ phase, which may explain the reduced proliferation of the Ishikawa cells. As shown in Figure 2D, the accumulation of these sub G_0_/G_1_ phase Ishikawa cells (*y* = 9.736 *x* − 10.545 *R*^2^ = 0.9016) following exposure to PCAE for 24 h implies that the Ishikawa cells may have undergone apoptosis.

### 2.4. Assessment of Changes in Mitochondrial Membrane Potential

**Figure 3:**
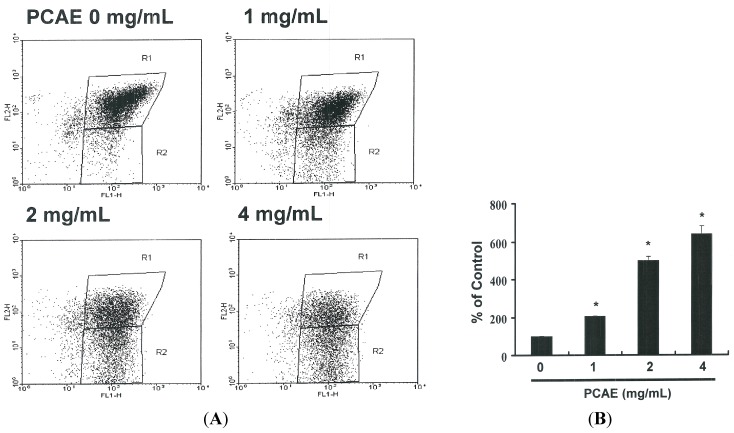

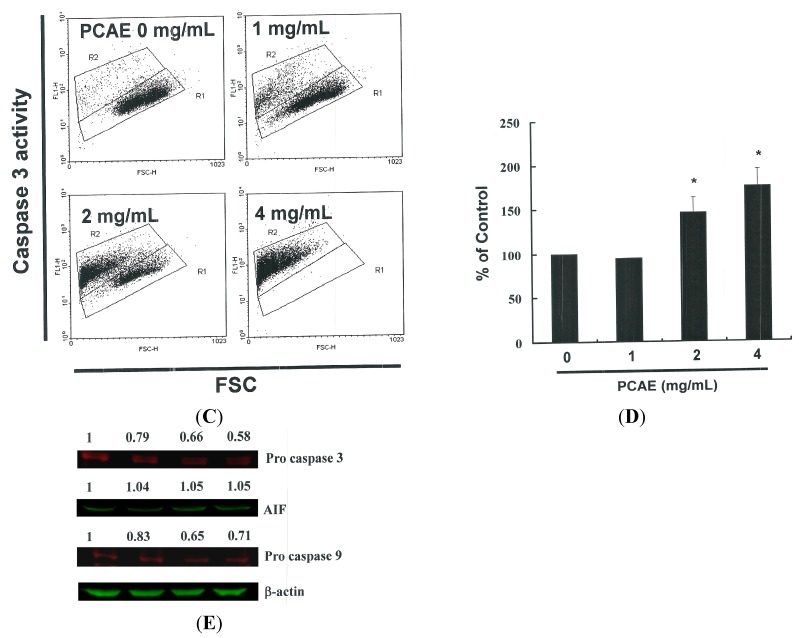
(**A**) Reduction of mitochondrial membrane potential (MMP) in Ishikawa cells by PCAE, as determined by JC-1 staining and detected by flow cytometry: MMP was shown to be significantly reduced in Ishikawa cells treated with PCAE (0, 1, 2, and 5 mg/mL); (**B**) Quantification by flow cytometry; (**C**) Caspase-3 activity in Ishikawa cells following treatment with PCAE for 24 h. Following treatment, the cells were harvested and labeled using FITC rabbit anti-active caspase-3; (**D**) Activation was quantified by flow cytometry; and (**E**) Cells were treated with PCAE for 24 h and procaspase-3, and -9, as well as AIF proteins were subsequently detected by Western blot analysis. All data are reported as the mean (±SEM) of at least three separate experiments. The * symbol in each group of bars indicates significant differences between PCAE treatment and control groups (*p* < 0.05).

The loss of mitochondrial membrane potential is a hallmark of apoptosis, coinciding with caspase activation. In non-apoptotic cells, JC-1 exists as a monomer in the cytosol (green) and accumulates as an aggregate in the mitochondria (red). Figure 3A presents typical FL-1/FL-2 dot plots associated with the JC-1 staining of Ishikawa cells with and without apoptosis. Untreated Ishikawa cells presented no signs of apoptosis and have red fluorescing J-aggregates. The green fluorescing monomers shown in the lower part of the figure indicate apoptotic cells (PCAE treatment using 1, 2, or 4 mg/mL). Figure 3B presents the percentages of apoptotic Ishikawa cells in various PCAE-treated groups as analyzed by flow cytometry. A decrease in mitochondrial membrane potential was observed at all dosage levels following treatment for 6 h. Specifically, treatment with 1 mg/mL PCAE led to a reduction of 2.06 *x* (*y* = 191.64 *x* − 117.23 *R*^2^ = 0.9673). A further increase in the concentrations of PCAE to 2 and 4 mg/mL led to reductions of 5.01 *x* and 6.41 *x*, respectively. These findings imply that PCAE significantly reduced the mitochondrial membrane potential of Ishikawa cells.

### 2.5. Induction of Apoptosis in Ishikawa Cells by PCAE via Caspase-3 Activation

Figure 3E presents the immunoblotting results for cellular proteins from Ishikawa cells treated with PCAE, which present a decrease in pro-caspase-3 and -9, but not in AIF. The relative band intensities of pro-caspase-3 and -9 were significantly lower in cells incubated with PCAE at concentrations of 1 to 4 mg/mL (Figure 3C). The results in Figure 3D suggest increased caspase-3 activation in Ishikawa cells (*y* = 27.92 *x* + 59.394 *R*^2^ = 0.8638) following PCAE treatment for 24 h. However, the results in Figure 2 and Figure 3 indicate that PCAE may mediate the survival of Ishikawa cells. Thus, we hypothesize that the proliferation of these cells was inhibited by pathways other than those of apoptosis.

**Figure 4:**
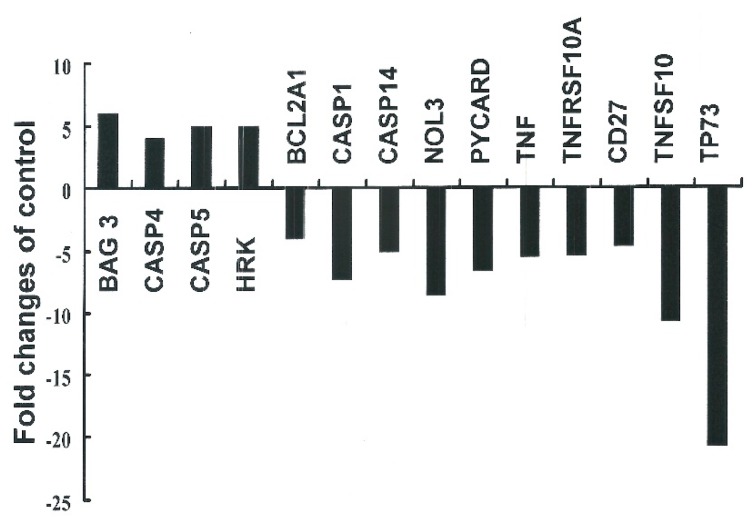
The focused panel gene expression profile of Ishikawa cells was studied using the RT^2^ Profilter^TM^ PCR array following four-hour exposure to vehicle (DMSO) or PCAE 2 mg/mL. Fold changes greater than two (up-regulation) or smaller than 0.5 (down-regulation) were considered substantial. Fold changes were calculated by dividing expression levels in PCAE-treated cells by expression levels in the vehicle-treated cells. BAG3: BCL-associated athanogene 3; CASP4: Caspase 4, apoptosis-related cysteine peptidase; CASP5: Caspase 5, apoptosis-related cysteine peptidase; HRK: Harakiri, BCL2 interacting protein; BCL2A1: BCL2-related protein A1; CASP1: Caspase 1, apoptosis-related cysteine peptidase; CASP14: Caspase 14, apoptosis-related cysteine peptidase, NOL3: Nuclear protein 3; PYCARD: PYD and CARD domain containing; TNF: Tumor necrosis factor, TNFRSF10A: Tumor necrosis factor receptor superfamily, member 10a; CD27: CD27 molecule, TNFSF10: Tumor necrosis factor receptor superfamily, member 10; TP73: Tumor protein p73.

Principal component analysis (PCA) revealed that the PCR-array data derived from PCAE-treated cells and DMSO-treated cells constituted two spatially-separated planes. This suggests that treatment with PCAE had a far greater impact on the gene expression profile than could be reasonably attributed to technical errors. We therefore divided the expression levels in PCAE-treated cells by those of the vehicle-treated cells and considered changes greater than two-fold to be substantial up-regulation and changes smaller than 0.5-fold to be down-regulation (Figure 4).

PC has been used in traditional medicine throughout Asia for the treatment of tumors, and many studies have sought to elucidate the mechanism underlying its anticancer activity [15].

In this study, PCAE demonstrated anti-tumor activity as well as the ability to induce apoptosis in EC (Ishikawa) cells. Our findings provide experimental evidence to support the contention that PCAE may irreversibly arrest EC cell growth. The results of mechanistic analysis led us to conclude that the inhibition of proliferation and the induction of apoptosis are both highly dependent upon the accumulation of PCAE in Ishikawa cells.

Apoptosis can be triggered by a wide variety of stimuli. To date, two major intracellular apoptosis signaling pathways have been identified: intrinsic and extrinsic. In both pathways, stress-mediated apoptosis is often triggered by a loss of mitochondrial function [16]. PCAE-mediated apoptosis was also explored using MTT viability and MMP assays, the results strongly suggest that PCAE-induced apoptosis follows an intrinsic pathway related to mitochondrial dysfunction.

Apoptotic stimuli trigger a family of cysteinyl aspartate-specific proteases (caspases) activation. It in turn initiates and executes the apoptotic pathways [17,18]. Caspases must undergo a proteolytic cleavage before converting to an active form during apoptosis. Caspase-3 is a key effector in the apoptosis pathway, amplifying the signal from initiator caspases (like caspase-9) and signifying full commitment to cellular disassembly [19]. Caspase-3 serves as a measure of early apoptosis pathway, whereas DNS fragmentation serves as an indicator of late apoptosis [20]. Cleaving caspase-3 initiates the cell death signals and the eventual induction of apoptosis. Thus, we surmise that PCAE-induced apoptosis is mediated through the activation of caspases. These results indicate that PCAE may delay cancer cell growth by apoptosis may via up-regulation of BCL-associated athanogene 3 (*BAG3*), *Caspase 4*, *Caspase 5* gene expression (Figure 4). BAG3 is constitutively present in few cell types, in which the protein appears to contribute to cell resistance to mechanical stress, cell survival, resistance to therapy, and/or motility and metastatization [21]. The caspase-1-like subfamily includes caspase-1, caspase-4 (TX/ICH-2/ICErelII), and caspase-5 (ICErelIII/TY) [22]. Caspase-4 and caspase-5 localize to the endoplasmic reticulum (ER) and may be activated by drugs that induce ER-stress induced apoptosis [23].

## 3. Experimental Section

### 3.1. Materials

*Pogostemon cablin* were a gift from Rich Fountain International Corp. MTT [3-(4,5-dimethylthiazol-2-yl)-2,5-diphenyltetrazolium bromide] and DMSO (dimethyl sulfoxide) were purchased from Sigma (St. Louis, MO, USA). DMEM (Dulbecco Modified Eagle Medium), FBS (fetal bovine serum), PBS (phosphate-buffered saline), sodium pyruvate, trypsin, and antibiotics were purchased from Gibco, BRL (Grand Island, NY, USA). PVDF (Polyvinylidene fluoride membrane) (Millipore, Billerica, MA, USA), and molecular weight marker were purchased from BioRad (Hercules, CA, USA). All other reagents and compounds were analytical grades.

### 3.2. PC Aqueous Extracts (PCAE) Preparation

The PC (400 g) was boiled with 200 mL distilled water at 100 °C for 4 h and total extract was evaporated under reduced pressure for 48 h to give 1.5 grams PCAE.

### 3.3. Cells

The Ishikawa cells were from ECACC (Sigma-99040201). The cells were maintained on culture dishes, in 90% (*v*/*v*) MEM with 2 mM l-glutamine and contain 1.5 g/L sodium bicarbonate with 10% (*v*/*v*) fetal bovine serum (FBS). The cells were cultured in an atmosphere containing 5% CO_2_ at 37 °C in an incubator.

### 3.4. Cell Proliferation Assay

The Ishikawa cells were seeded 5000 cells/well into 96-well culture plates. Those cells were added with 0, 1, 2 and 4 mg/mL PCAE for 24 to 72 h. The MTT dye (1 mg/mL) was treated to each well for the at least 4 h of treatment. The reaction was stopped with DMSO addition, and OD_540_ (optical density) was measured by a multi-well plate reader. Background absorbance of the medium in the absence of cells was subtracted. All samples were assayed at least in triplicate, and the mean for each experiment was calculated. Results were expressed as a percentage of control, which was considered 100%. Each assay was carried out in triplicate and the results were expressed as the mean (±SEM).

### 3.5. Measurement of Apoptosis

The Ishikawa cells were first seeded in 6-well culture plates (Orange Scientific, Braine-l’Alleud, Belgium). Following treatment with PCAE for four hours, the Ishikawa cells were harvested. The cells were centrifuged (the supernatant discarded) and resuspended/incubated with 1× annexin-binding buffer: 5 μL of annexin V-FITC (BD Pharmingen, San Jose, CA, USA) and 1 μL of 100 μg/mL PI working solution for 15 min at room temperature. After the incubation period, the stained cells were analyzed by FACSCalibur flow cytometry (BD, Franklin Lakes, NJ, USA). Data was analyzed using WinMDI 2.8 free software (BD).

### 3.6. Caspase-3 Activity Assay

The caspase activity was assessed by the FITC rabbit anti-active caspase-3 (BD Pharmingen). The Ishikawa cells were treated with PCAE of 0, 1, 2 and 4 mg/mL for 1 day. The activity of caspase was detected and inspected by the FACSCalibur flow cytometry (BD). Data was analyzed by WinMDI 2.8 free software (BD).

### 3.7. Cell Cycle Analysis

We used the fluorescent nucleic acid dye propidium iodide (PI) to identify the proportion of cells in each of the three interphase stages of the cell cycle. The cancer cells were treated with PCAE for 24 h, and then harvested and fixed in 1 mL cold 70% ethanol overnight at −20 °C. DNA was stained in PI/RNaseA solution and the DNA content was detected using FACSCalibur flow cytometry and the data was analyzed using WinMDI 2.8 free software (BD).

### 3.8. Western Blot Assay

A total of 50 to 75 μg of proteins were separated by 10%–12% SDS-PAGE, and transferred to PVDF membranes (Millipore). The PVDF were blocked with blocking buffer (Odyssey, Danbury, CT, USA) overnight, and incubated with anti-β-actin (Sigma-Aldrich, St. Louis, MO, USA), anti-caspase 3, anti-AIF and anti-caspase 9 (Santa Cruz BioTechnology, Dallas, TX, USA) antibodies for 90 to 120 min. The blots were washed and incubated with a second antibody (IRDye Li-COR, Lincoln, NE, USA) at a 1/20,000 dilution for 30–45 min. The antigens were then visualized using a near infrared imaging system (Odyssey LI-COR) and data was analyzed by Odyssey 2.1 software.

### 3.9. Gene Expression Profiling (GEP)

Briefly, the cells untreated or treated with PCAE for 4 h, were harvested and total RNA was isolated utilizing an RNasey kit (QIAGEN, Redwood City, CA, USA) as described by the manufacturer. The focused panel of genes was analyzed by RT^2^ Profiler™ PCR Array (PAHS-012Z, QIAGEN).

### 3.10. Statistical Analysis

All data were reported as the mean (±SEM) of at least three separate experiments. A *t*-test or one-way ANOVA with *post-hoc* test was employed for statistical analysis, with significant differences determined as *p* < 0.05.

## 4. Conclusions

The effects of PCAE and related compounds in cancer therapy and prevention are already well established. Their potential to serve as antitumor agents also warrants further investigation.
